# Grapevine Pinot Gris Virus Is Present in Different Non-*Vitis* Hosts

**DOI:** 10.3390/plants11141830

**Published:** 2022-07-12

**Authors:** Emese Demian, Nikoletta Jaksa-Czotter, Eva Varallyay

**Affiliations:** Genomics Research Group, Department of Plant Pathology, Institute of Plant Protection, Hungarian University of Agriculture and Life Sciences, 2100 Godollo, Hungary; emese.demian@gmail.com (E.D.); jaksa-czotter.nikoletta@uni-mate.hu (N.J.-C.)

**Keywords:** plant virus, grapevine, GPGV, survey, non-*Vitis*, alternative host, asymptomatic variant

## Abstract

Grapevine Pinot gris virus (GPGV) was described in Italy using a metagenomic approach: next-generation sequencing of the virus-derived small RNAs. Since that time, it has been reported all over the world. The presence of GPGV is associated with grapevine disease, but most of the time, the disease is asymptomatic. Although the host range of this virus has not been investigated, it has been found in the non-*Vitis* hosts, *Silene latifolia* and *Chenopodium album*. We investigated the presence of GPGV in grapevine and other plant species growing as weeds in the vineyard. Using RT-PCR, we identified GPGV in seven non-*Vitis* hosts: *Ailanthus*, *Asclepias*, *Crataegus*, *Fraxinus*, *Rosa*, *Rubus*, and *Sambucus*. In the case of *Rosa* and *Rubus*, this finding was supported by Northern blot detection of the virus. GPGV strains in non-*Vitis* hosts belong to the asymptomatic clade, and are clustered according to their original geographic locations. The presence of GPGV in species other than grapevine shows that besides well-known vector and propagating material-based infections, other possible entry sites for the virus can exist, which have to be taken into consideration when developing reliable regulation strategies.

## 1. Introduction

Grapevine Pinot gris virus (GPGV), a member of the trichovirus (Betaflexiviridae) genus, was first identified in vineyards in Trentino, Italy, by small RNA (sRNA) high-throughput sequencing (HTS) [1] as a causative agent of grapevine leaf mottling and deformation (GLMD), a new grapevine disease reported since 2003. The virus is widespread, and has been reported in 58 countries on five continents (EPPO, updated 21 February 2022). The disease seems to be confined to Italy, and in some sensitive cultivars since the beginning, its Italian origin have been suspected. With a widespread distribution described in Slovakia [2], Moravia [3], and Hungary [4,5], its origin in this part of Central-Eastern Europe was hypothesized [6,7]. The latest phylogeographic reconstruction analysis revealed that GPGV could have originated from Asia, most probably China [8], from where it was introduced to Europe. This European population served as a base for worldwide distribution, possibly by trading symptom-free but GPGV-infected propagating material [8]. It is transmitted via grafting [9]. GPGV was found in the bodies of the eriophyid mite *Colomerus vitis*, and was transmitted to healthy vines through mite infestation [10], revealing another mode of entry for infection. Furthermore, GPGV has been detected in two herbaceous hosts, *Silene latifolia* subsp. *Alba* (Mill) and *Chenopodium album* L. [11].

GPGV variants show low genetic diversity (a sequence identity of higher than 97%) [12], but clear differences between the three lineages in terms of virulent and latent variants have been reported [9,13]. A risk assessment of GPGV has not yet resulted in regulation, and without the regular testing of stock cultivars and with the absence of symptoms on rootstocks, its spread cannot be reliably controlled [14].

In a survey of endemic plants and invasive weeds surrounding the vineyards, GPGV could be detected in common milkweed, *Rubus*, *Rosa*, and even in *Fraxinus* [15]. In the current work, we investigated the presence of GPGV in non-*Vitis* hosts in more detail than in our initial observation, to gain information regarding the possible wider host range of GPGV.

## 2. Results

### 2.1. GPGV Is Present in Plants Species Other Than Grapevines

**Figure 1:**
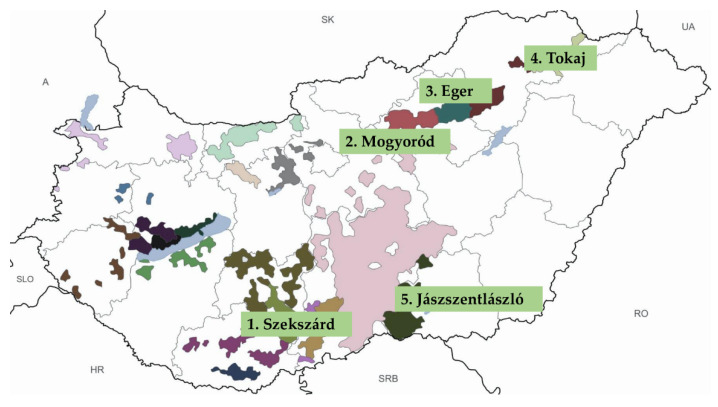
Location of sample collection sites in different vine growing regions of Hungary.

Surveying vineyards and grapevine rootstock plantations, we detected GPGV infection in distinct parts of the country [4,5]. For this study, five GPGV-infected vineyards showing no GLMD symptoms situated in different wine-growing regions of Hungary were selected (Figure 1).

Vineyards at Szekszard, Eger, Tokaj (Figure S1), and Jaszszentlaszlo were regularly maintained, and the weeds were removed regularly. The vineyard at Mogyorod had been neglected for more than a decade, without any weed removal (Figure S2). To check the presence of GPGV in species other than grapevine, weeds growing within the vineyard were sampled (Table 1): *Chenopodium album* (Figure S3), *Asclepias syriaca*, *Ailanthus* sp., *Rosa canina*, *Crataegus* sp., *Sambucus* sp. (Figures S4 and S5), *Rubus* sp. (Figure S6), and *Fraxinus* sp. (Figure S7).

**Figure 2:**
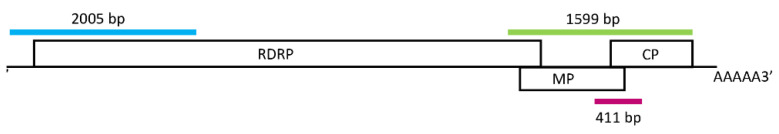
Schematic representation of cloned regions of GPGV, indicating size and position on GPGV genome; 411 bp amplicon was generated using primers described by Glasa et al. [2].

Though the leaves of the sampled plants had different spots, chlorosis, and malformations, none of them showed any specific symptoms resembling GLMD. The presence of GPGV was tested using RT-PCR. First, diagnostic primers from Glasa et al. [2] were used to check for the presence of the virus. In the case of the positive samples, we amplified and cloned a 2005 nt portion of the viral genome from the 5′ end of the virus, including partial 5′ UTR and replicase, and a 1599 nt portion from the MP/CP coding region (Figure 2, Table S1).

**Figure 3:**
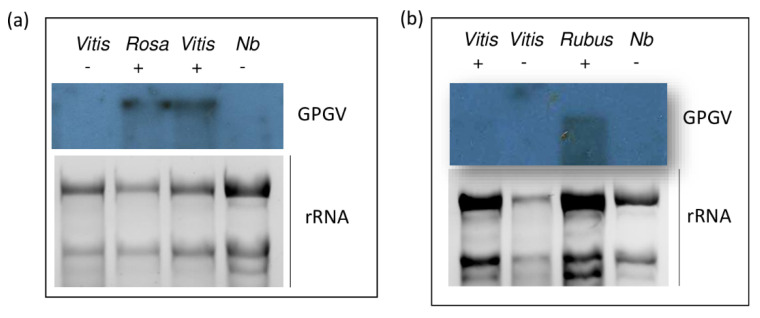
Northern blot detection of GPGV in samples collected at (**a**) Eger and (**b**) Tokaj. Vitis indicates RNA extracted from grapevine, Rosa from rose, and Rubus from Rubus, while Nb indicates RNA extracted from Nicotiana benthamiana used as a negative control. – and + indicate RT-PCR results for GPGV in same samples. Radiolabeled probe was used for hybridization. rRNA on EtBr-stained gel served as loading control.

In some cases, we failed to amplify the targeted amplicon. In C. album, we could only amplify the 411 bp product, possibly because of the low virus titer in the plant. At Jaszszentlaszlo, we could amplify GPGV only by using cDNA prepared with oligodT primer, even from grapevine, and we failed to amplify the 5′ part of the virus, suggesting its low titer in the plants at this site. We had the same difficulty with woody species at Mogyorod. Although we could amplify GPGV using random primer-produced cDNA, we could not amplify the 5′ part of the virus. Our attempt to detect the virus with Northern blot was successful in only two cases, the Rosa sample originating from Eger and the Rubus sample originating from Tokaj, suggesting a higher virus titer in these two cases (Figure 3).

### 2.2. GPGV Variants Are Very Similar and Cluster According to the Sample Location

The amplified parts of the virus with different origins were cloned and Sanger sequenced. The sequences were analyzed at both the nucleotide and amino acid level. Sequences of the variants were compared to the reference genome in the case of grapevine-originated samples. We compared the alternative host-derived GPGV isolates to the Silene-originated GPGV genome in GenBank (Table 2 and Table 3).

**Figure 4:**
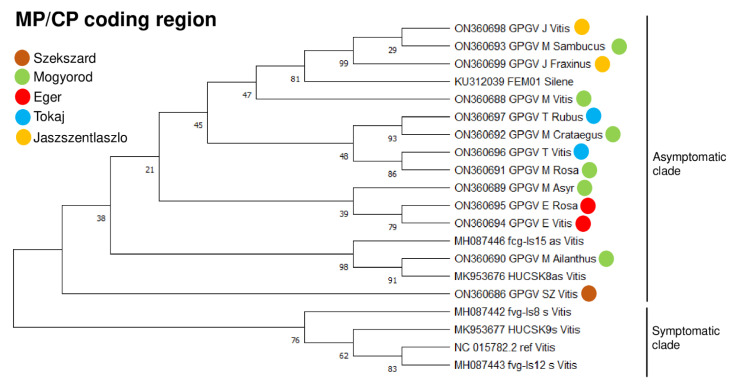
Evolutionary analysis of MP/CP coding region using the maximum likelihood method. Evolutionary history was inferred using the maximum likelihood method and the Tamura–Nei model. Bootstrap consensus tree inferred from 1000 replicates is taken to represent evolutionary history of analyzed taxa. Branches corresponding to partitions reproduced in less than 50% bootstrap replicates are collapsed. Percentage of replicate trees in which associated taxa clustered together in bootstrap test (1000 replicates) is shown next to branches. Evolutionary analyses were conducted in MEGA11. Colored circles indicate geographical origins of strains. HUCSK8as and HUCSK9s are asymptomatic and symptomatic GPGV strains described in Hungary [5]. Ref is the GPGV reference genome from grapevine; FEM01 strain was described from Silene [11]. Symptomatic (s) and asymptomatic (as) GPGV isolates Fvg-is-8_s, 12_s, and 15_as were described by Tarquini et al. [12].

**Figure 5:**
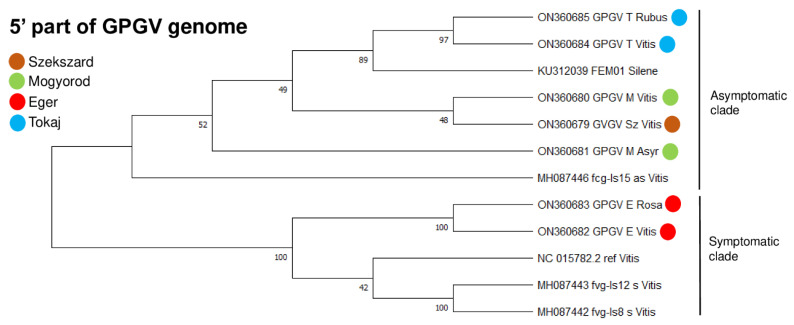
Evolutionary analysis of 5′ nucleotide region of GPGV genome by the maximum likelihood method. Evolutionary history was inferred by using the maximum likelihood method and the Tamura–Nei model. Bootstrap consensus tree inferred from 1000 replicates is taken to represent evolutionary history of analyzed taxa. Branches corresponding to partitions reproduced in less than 50% bootstrap replicates are collapsed. Percentage of replicate trees in which associated taxa clustered in bootstrap test (1000 replicates) is shown next to branches. Evolutionary analyses were conducted in MEGA11. Colored circles indicate geographical origins of strains. HUCSK8as and HUCSK9s are asymptomatic and symptomatic GPGV strains described in Hungary [5]. Ref is GPGV reference genome from grapevine; FEM01 strain was described from Silene [11]. Symptomatic (s) and asymptomatic (as) GPGV isolates Fvg-is-8_s, 12_s, and 15_as were described by Tarquini et al. [12].

A comparison of the GPGV sequences shows a very high rate of identity to the reference genomes when we compared either the nucleotide sequences of the viral genome or the amino acid sequences of the encoded proteins. Identities were higher than 97% in all investigated cases (sequence of the replicase, movement protein, or coat protein). The only exception was when we compared the movement protein-coding capacity of grapevine isolates to the reference genome. The phylogenetic relationship of the GPGV isolates was investigated to find out their evolutionary relationship (Figure 4 and Figure 5). From the phylogenetic analysis of the MP/CP coding region, it is obvious that at most of the maintained vineyards (Eger, Tokaj, and Jaszszentlaszlo), GPGV variants from different hosts (grapevine and other species) clustered together according to their geographical origins, suggesting an on-site infection (Figure 4).

GPGV variants at the old, neglected vineyard at Mogyorod were very diverse, suggesting a longer evolution of the virus in these woody perennial hosts. This longer time, extending for several years, allowed the virus to incorporate several mutations within the viral genome and diverge. In *C. album*, we could only amplify the 411 bp region of the virus. The phylogenetic tree, including this isolate, shows that it does not cluster with the grapevine isolate (SZ_Vitis) from the same geographical origin (Figure S8). Phylogenetic analysis based on the 5′ UTR and the RdRp coding part of the viral genome was also performed (Figure 5).

According to this analysis, isolates that originated from Tokaj and Eger were clustered according to their place of origin, while the sequences that originated from Mogyorod clustered distantly, showing the same trend that was found based on the MP/CP region. However, in the latter analysis, Sz_Vitis, M_Asyr, E_Rosa, and E_Vitis isolates clustered differently than in the MP/CP-based analysis, suggesting a possible recombination event during their evolution. Most of the variants clustered together with the asymptomatic GPGV variants, as expected based on the latent presence of the virus on grapevine. However, it is very surprising that GPGV variants from Eger (both Vitis and Rosa variants, investigating the 5′ part of the genome) clustered together with the symptomatic reference strain of the virus.

An investigation of the presence of symptomatic and asymptomatic clade-specific SNPs revealed that although the above-mentioned E_Vitis and E_Rosa variants clustered together with the symptomatic variants, they did not have either the early stop codon at 6670 or the symptomatic variant-specific SNPs at 6400 and 6593 in the movement protein (Figure S9). It is interesting to note that the polymorphism at 6593 is not present in HUCSK9s, the only symptomatic GPGV variant detected in Hungary to date [5]. Unfortunately, we could not check for the presence of SNP at 1922, one of the symptomatic variants connected to SNP variation in the replicase, because our primers were just positioned at that site (Figure S10). The other replicase positioned polymorphism (1360) was the symptomatic version in both E_Rosa and E_Vitis. Tarquini and colleagues identified five more SNPs in the movement protein-coding region of the virus [12], which are different between the symptomatic clades β and γ. An investigation of these polymorphisms showed that at 5588 and 6659, our isolates contained the SNP specific for the asymptomatic variants (Figure S11). At SNP6320, E_Rosa, E_Vitis, and M_Asyr variants contained the variant specific for the symptomatic β clade coding for Asn instead of Ser or Thr (in the reference strain). At SNP6452, most of the Hungarian strains encode CTA (encoding Leu), but T_Rubus, M_Crataegus, M_Vitis, M_Rosa, and T_Vitis variants encode CCA (encoding Pro), similar to γ clade, while HUCSK9s contains another variant, TCA (encoding Ser). SNP6392 seems very diverse; besides TCT (encoding Ser), which is present in β clade, TTT (Phe), CTT (Leu), and CCT (encoding Pro in the E_Rosa variant) are also present.

## 3. Discussion

GPGV was detected in all of the examined vineyards, and besides grapevine, its presence was detected in seven non-*Vitis* hosts: *Ailanthus*, *Asclepias*, *Crataegus*, *Fraxinus*, *Rosa*, *Rubus*, and *Sambucus.* The presence of the virus was not accompanied by typical GPGV-specific symptoms, either on grapevine or on weeds. There are different theories to explain the symptomatology of GPGV. One explanation is that there are symptomatic and asymptomatic variants, which we will discuss later. Investigating virus variants together with virus concentrations led to another hypothesis: that the virus titer can influence the development of symptoms [13]. In that work, qRT-PCR based on SYBR Green chemistry was performed with optimized internal controls, glyceraldehyde-3-phosphate dehydrogenase, and cytochrome oxidase, as the most stably expressed genes. In contrast, by investigating Spanish vineyards with the TaqMan method and using the phosphoenolpyruvate carboxylase gene as an internal control, no correlation between virus concentration and symptom development was found [16]. In the case of *Rosa* and *Rubus*, we could support the RT-PCR result, and could detect the presence of GPGV using a less sensitive Northern blot assay, suggesting a relatively higher virus concentration in these two samples. In *C. album* we could only amplify a 411 bp product from the MP/CP region, while at Jaszszentlaszlo, we could only amplify GPGV if we used oligodT primers for cDNA synthesis. We could not amplify the 5′ part of the viral genome either in J_Fraxinus and J_Vitis, or in M_Ailanthus, M_Crataegus, or M_Sambucus, which are woody hosts at Mogyorod. Such failure in these cases suggests that, in both *C. album* and the three woody hosts, the virus concentration was low, below the sensitivity threshold of the method. It could also be possible that in these hosts, a variant with a mutation in the primer coding region was present, leading to an inability to detect them.

The other hypothesis for the symptomatic and asymptomatic behavior of GPGV is the existence of symptomatic and asymptomatic variants of the virus. The most striking difference between the two variants is the polymorphism at the end of the MP (6670 in the reference genome). The symptomatic variants encode an early stop codon, resulting in a protein that is shorter by six amino acids [9]. In addition to this well-described polymorphism, four additional ones leading to amino acid changes in the encoded proteins, two in the replicase (1360 and 1922 position in the reference genome) and two in the movement protein (6400 and 6593 in the reference genome), were identified [12]. An investigation of the symptomatic variant-specific SNPs revealed that most of the strains characterized in this study had asymptomatic variant-specific SNPs. In contrast, E_Vitis and E_Rosa had symptomatic variant-specific SNPs in several places, and they clustered together with the symptomatic reference strains, although they lacked the early stop codon in their MP coding region.

A comparison of the sequences of GPGV variants in different isolates showed very high identity, higher than 97% in all but one case. When we compared the sequences of the grapevine-originated GPGV isolates to the reference genome, the identity on the amino acid level was only 95.39–96.21%. The reason for this is the presence of the longer MP coding region, characteristic of asymptomatic variants, in the investigated grapevine isolates.

Phylogenetic analysis of the 5′ part and the MP/CP region of the GPGV strains showed that the GPGV variants clustered according to the vineyard and not the host, suggesting an on-site infection. The shorter MP variant associated with the presence of symptoms was not found either in grapevine or in weeds, suggesting a widespread presence of the latent variant.

GPGV variants at the old, neglected vineyard clustered distantly and not together. It is possible that GPGV infection at this place happened a longer time ago, allowing for a longer evolution and spread of GPGV within these perennial hosts, allowing the virus to incorporate several mutations and diverge.

The host range of a virus is determined by several factors, but it is highly dependent on the virus vector. *Colomerus vitis*, the proven vector of GPGV, is monophagous and known to feed only on grape [10]. In line with the previous report of GPGV-infected *S. latifolia* and *C. album* [11], we show that GPGV can infect not just grapevine, and we agree that the presence of a polyphagous vector that assists in virus transmission between hosts can be anticipated.

The presence of GPGV in a vineyard’s neighboring woody or perennial hosts suggests that GPGV may in fact be endemic in Eastern Europe. Although its probable center of origin center is located in Asia, it is suggested that an intercontinental jump to Europe in the middle of the 20th century occurred [8]. Since that time, GPGV could have become widespread in Europe and survived not only in grapevines, but in the natural flora near vineyards.

## 4. Materials and Methods

### 4.1. Plant Material and Sample Preparation

Leaf samples were collected at 5 regions in Hungary (Figure 1) from grapevines and neighboring plants (Table 1) in May–August, 2015–2017. RNA was extracted from the grapevines and from rose using an optimized CTAB-based method [17], while a phenol-chloroform extraction method was used for the other plants [18]. Total nucleic acid extracts obtained were stored at −70 °C until use.

### 4.2. Virus Diagnostics via RT-PCR

RNA extracts of plants were used as templates for cDNA synthesis using random or oligodT with the RevertAid First Strand cDNA Synthesis Kit (Thermo Fisher Scientific, Waltham, MA, USA). The presence of GPGV was checked using diagnostic primers [2] amplifying a 411 bp part of MP-CP. For a sequence comparison, we used cDNA synthesized from the RNA of individuals as a template in the PCR, using Q5 Hot Start High-Fidelity DNA Polymerase (New England Biolabs, Ipswich, MA, USA), and amplified a 2005 bp product from the 5′ part (5′ UTR and partial RdRp) and a 1600 bp product from the 3’ MP-CP coding region (Table S1). PCR products were purified using a GeneJET1.2/blunt Gel Extraction Kit (Thermo Fisher Scientific), cloned into the pJET vector (Thermo Fisher Scientific), and sequenced. Sequences were deposited in GenBank (accession numbers: ON360679-ON360699).

### 4.3. Virus Detection via Northern Blot

For Northern blot analysis, 2–4 µg of total RNA was separated on formaldehyde/1.2% agarose gels and blotted to Nytran NX membrane (GE Healthcare, Chicago, IL, USA) via the capillary method using 20× SSC. Hybridization was performed at 65 °C in Church buffer (0.5 M phosphate buffer, pH 7.2, containing 1% BSA, 1 mM EDTA, and 7% SDS) overnight with the appropriate radioactively labeled probe, washed for 5 min in 2× SSC and 0.1% SDS, and incubated for 15 min in 0.5× SSC and 0.1% SDS at the hybridization temperature and exposed to X-ray film. Virus-specific P32-labeled DNA probes were prepared using the Thermo Scientific Decalabel DNA labeling kit.

### 4.4. Phylogenetic Analysis

For the SNP and phylogenetic analysis of viral sequences, we used Geneious Prime 2022.0.2 (https://www.geneious.com) and MEGA11 [19]. The viral sequences used for alignments were cut to equal lengths and aligned using MUSCLE embedded in MEGA11. The evolutionary history was inferred using the maximum likelihood method and the Tamura–Nei model [20]. The bootstrap consensus tree inferred from 1000 replicates [21] was taken to represent the evolutionary history of the analyzed taxa. Branches corresponding to partitions reproduced in less than 50% of the bootstrap replicates were collapsed. The percentage of replicate trees in which the associated taxa clustered together in the bootstrap test (1000 replicates) are shown next to the branches [21]. Initial trees for the heuristic search were obtained automatically by applying the neighbor-join and BioNJ algorithms to a matrix of pairwise distances estimated using the Tamura–Nei model, and then selecting the topology with superior log likelihood value.

## 5. Conclusions

In our work, we show that GPGV, a widespread virus infecting grapevine, can also be present in other species near vineyards. GPGV was proved to be transmitted via grafting and via a monophagous vector. Its presence in alternative hosts suggests the possibility of transmission via a polyphagous vector, which remains to be found and characterized.

The presence of GPGV in alternative hosts has importance in virus epidemiology. There is no official regulation dealing with GPGV, but this fact has to be kept in mind when making decisions regarding its regulation. This could prevent it from spreading by transport and exchange between these possibly infected alternative hosts.

## Figures and Tables

**Table 1:** Basic information of sampled plants and GenBank accession numbers of deposited GPGV sequences.

Sample Collection Site	Host/Cultivar	Date of Sampling	Strain	GPGV Amplified Region	
				5′ UTR and RdRp Region	3′ UTR and MP-CP Region
Szekszard	grapevine/Kadarka	May 2015	Sz_Vitis	ON360679	ON360686
	*Chenopodium album*		Sz_Calbum	¯	ON360687 *
Mogyorod	grapevine/Furmint	June 2016	M_Vitis	ON360680	ON360688
	*Asclepias syriaca*		M_Asyr	ON360681	ON360689
	*Ailanthus* sp.		M_Ailanthus	¯	ON360690
	*Rosa canina*		M_Rosa	¯	ON360691
	*Crataegus* sp.		M_Crataegus	¯	ON360692
	*Sambucus* sp.		M_Sambucus	¯	ON360693
Eger	grapevine/Kadarka	May 2016	E_Vitis	ON360682	ON360694
	*Rosa canina*		E_Rosa	ON360683	ON360695
Tokaj	grapevine/Furmint	June 2015	T_Vitis	ON360684	ON360696
	*Rubus* sp.		T_Rubus	ON360685	ON360697
Jaszszentlaszlo	grapevine	August 2017	J_Vitis	¯	ON360698
	*Fraxinus* sp.		J_Fraxinus	¯	ON360699

* Only a 411 bp coding region.

**Table 2:** Similarity of sequenced 5′ parts of GPGV strains to reference genome. Sequences from grapevine were compared to grapevine-originated reference, while sequences from weeds were compared to full GPGV genome originated from Silene.

Similarity to Reference							
Similarity to NC_015782.2_Vitis				Similarity to KU312039_Silene			
Strain	GB Accession	% nt Level	% aa Level	Strain	GB Accession	% nt Level	% aa Level
**Sz_Vitis**	ON360679	97.16	97.66				
**M_Vitis**	ON360680	97.21	97.34	**M_Asyr**	ON360681	97.36	98.28
**E_Vitis**	ON360682	97.91	98.28	**E_Rosa**	ON360683	97.91	97.66
**T_Vitis**	ON360684	97.16	97.19	**T_Rubus**	ON360685	96.86	98.44

**Table 3:** Similarity of sequenced MP/CP coding part of GPGV strains to reference genome. Sequences from grapevine were compared to grapevine-originated reference, while sequences from weeds were compared to full GPGV genome originated from Silene.

Similarity to Reference									
Similarity to NC_015782.2_Vitis					Similarity to KU312039_Silene				
Strain	GB Accession	% nt Level	MP % aa Level	CP % aa Level	Strain	GB Accession	% nt Level	MP % aa Level	CP % aa Level
**Sz_Vitis**	ON360686	96.94	96.21	99.49	**Sz_Calbum**	ON360687	97.57		97.08
**M_Vitis**	ON360688	96.62	95.39	98.46	**M_Asyr**	ON360689	97.81	98.13	97.95
					**M_Ailanthus**	ON360690	97.31	97.33	98.46
					**M_Rosa**	ON360691	98.38	98.93	98.97
					**M_Crataegus**	ON360692	97.94	98.67	98.46
					**M_Sambucus**	ON360693	98.38	99.47	98.97
**E_Vitis**	ON360694	97.06	95.66	100.00	**E_Rosa**	ON360695	97.81	97.87	98.97
**T_Vitis**	ON360696	97.12	96.21	99.49	**T_Rubus**	ON360697	97.94	98.67	98.46
**J_Vitis**	ON360698	96.75	95.66	99.49	**J_Fraxinus**	ON360699	98.38	99.47	98.97

## Data Availability

Sequences of the virus variants are available from the NCBI GenBank under the following accession numbers: ON360679–ON360699.

## Supplementary Materials

The following supporting information can be downloaded at https://www.mdpi.com/article/10.3390/plants11141830/s1: Demian et al._Plants_2022_S1, containing Figure S1: Field view of sampled maintained vineyards at Szekszard, Eger, and Tokaj; Figure S2: Field view of sampled neglected vineyard at Mogyorod; Figure S3: Symptoms of *Chenopodium album* collected at Szekszard that proved to be GPGV-infected; Figure S4: Symptoms of sampled plants collected at Mogyorod that proved to be GPGV-infected; Figure S5: Symptoms of *Rosa canina* collected at Eger that proved to be GPGV-infected; Figure S6: Symptoms of sampled *Rubus* sp. that proved to be GPGV-infected; Figure S7: Symptoms of *Fraxinus* sp. collected at Jaszszentlaszlo that proved to be GPGV-infected; Figure S8. Evolutionary analysis of 411 bp part of MP/CP coding region of GPGV by maximum likelihood method; Figure S9: Polymorphism in movement-coding region of asymptomatic and symptomatic GPGV variants; Figure S10: Polymorphism in replicase-coding region of asymptomatic and symptomatic GPGV variants; Figure S11: Polymorphism in movement protein-coding region of asymptomatic and symptomatic GPGV variants in position differentiating between clades β and γ. Table S1: Sequences of PCR primers used for virus detection, with their appropriate references.
