# Supplementary material for: Grapevine Pinot Gris Virus Is Present in Different Non-Vitis Hosts
Source: Plants (Basel). 2022 Jul 12;11(14):1830. doi: 10.3390/plants11141830 (PMC9315623; doi:10.3390/plants11141830)
Supplement: Supplementary file 1 [file plants-11-01830-s001.zip › Demian et al_Plants_2022_S1.pdf]

Supporting S1 to

Grapevine Pinot gris virus is present in different non-*Vitis* hosts

Emese Demian, Nikoletta Jaksa-Czotter and Eva Varallyay*

* Correspondence: varallyay.eva@uni-mate.hu

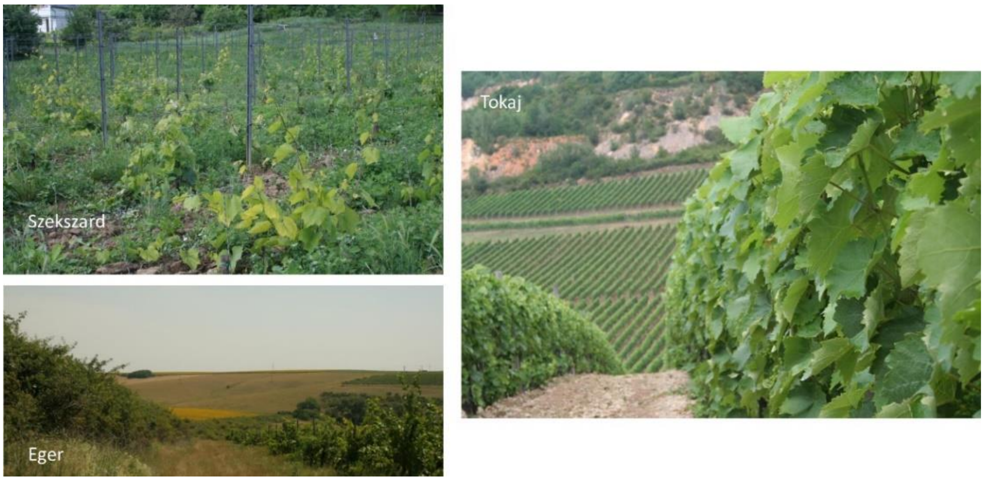

Figure S1. Field view of sampled maintained vineyards at Szekszard, Eger, and Tokaj.

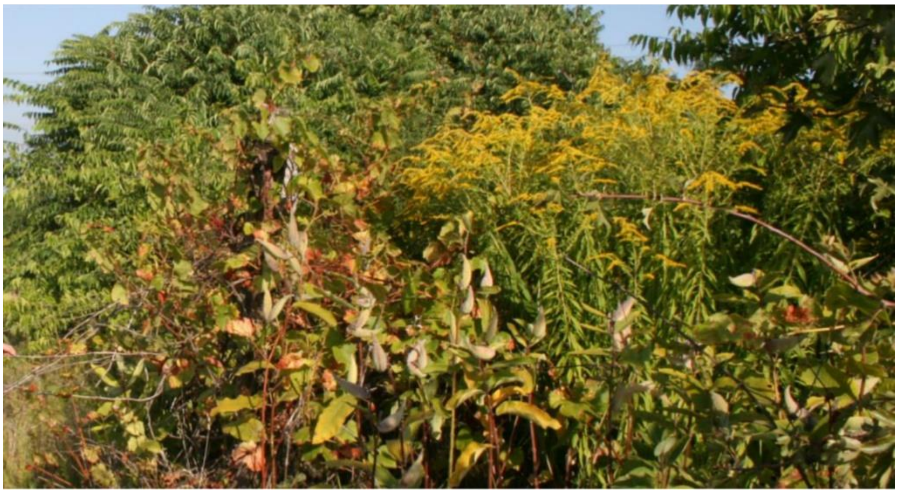

Figure S2: Field view of sampled neglected vineyard at Mogyorod.

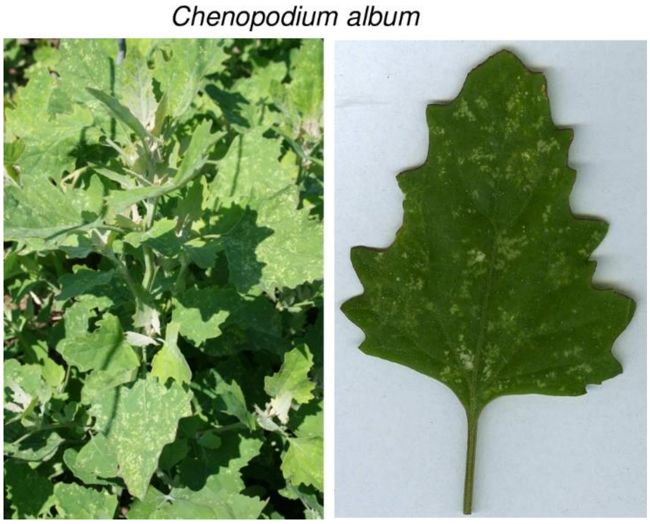

Figure S3: Symptoms of Chenopodium album collected at Szekszard that proved to be GPGV infected.

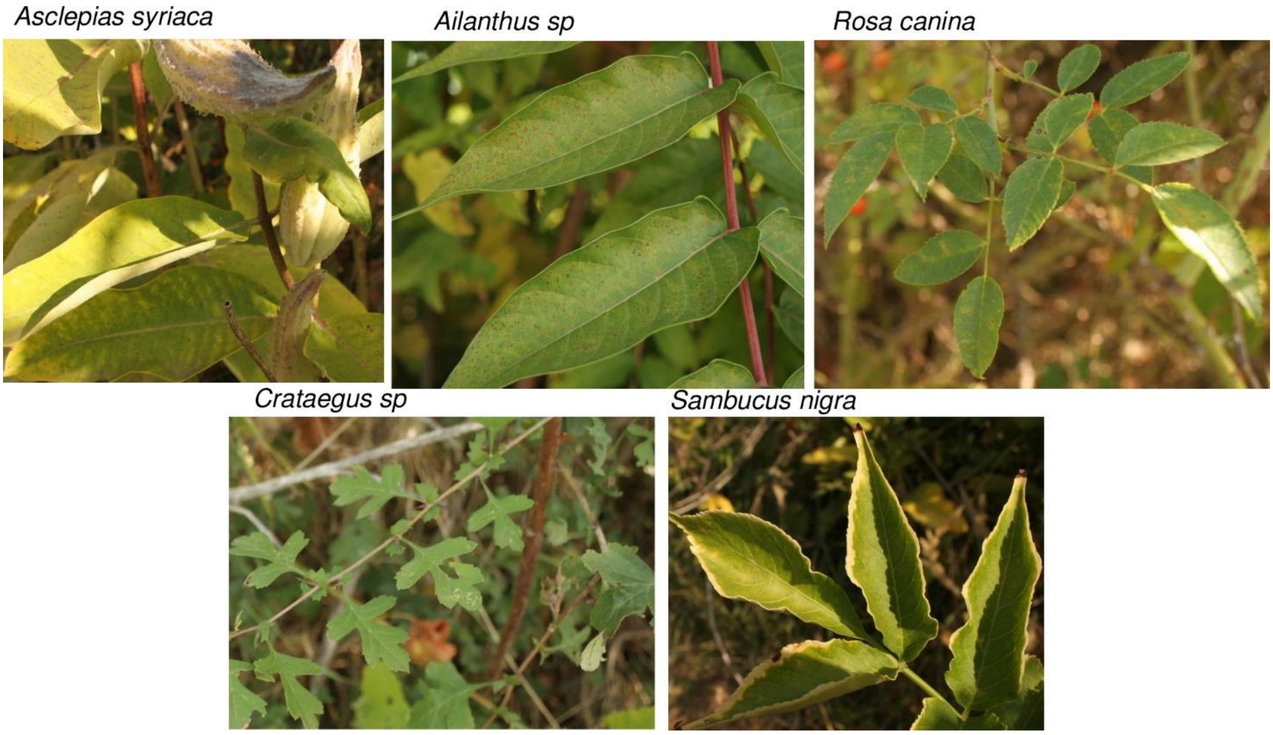

Figure S4: Symptoms of sampled plants collected at Mogyorod that proved to be GPGV infected.

Rosa canina

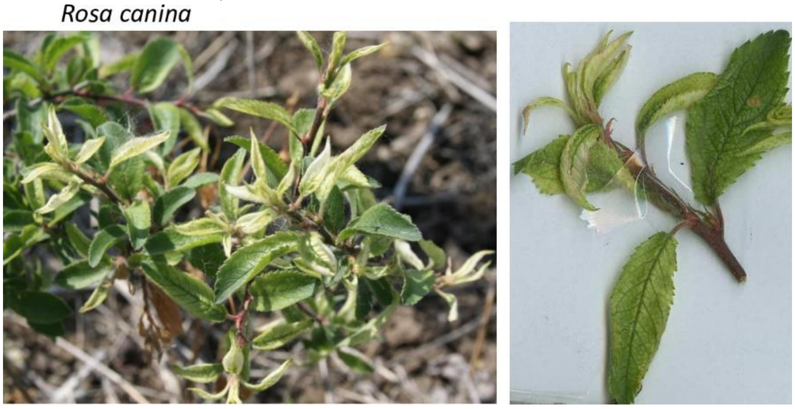

Figure S5: Symptoms of Rosa canina collected at Eger that proved to be GPGV infected.

Rubus sp

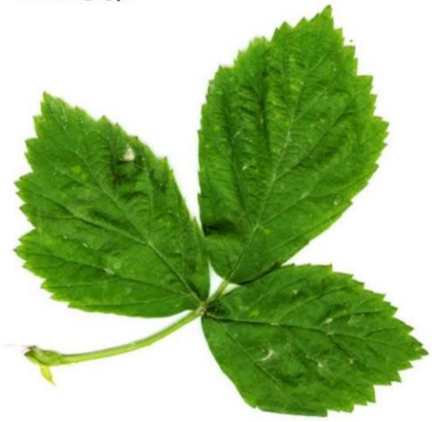

Figure S6: Symptoms of sampled Rubus sp. that proved to be GPGV infected.

Fraxinus sp

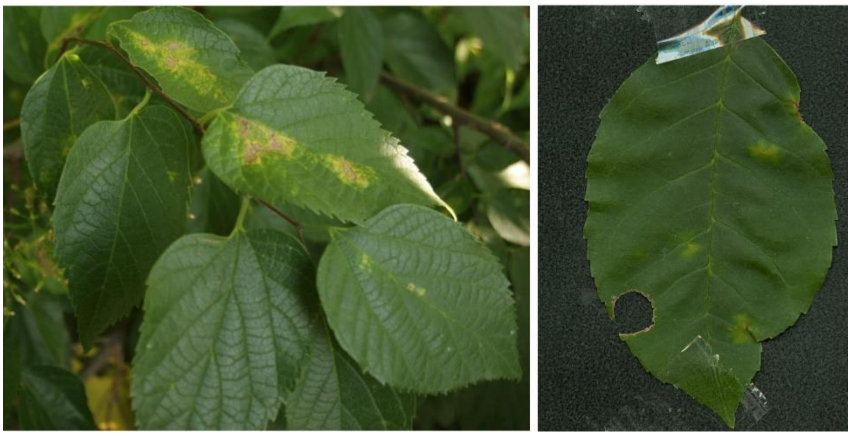

Figure S7: Symptoms of Fraxinus sp. collected at Jaszszentlaszlo that proved to be GPGV infected.

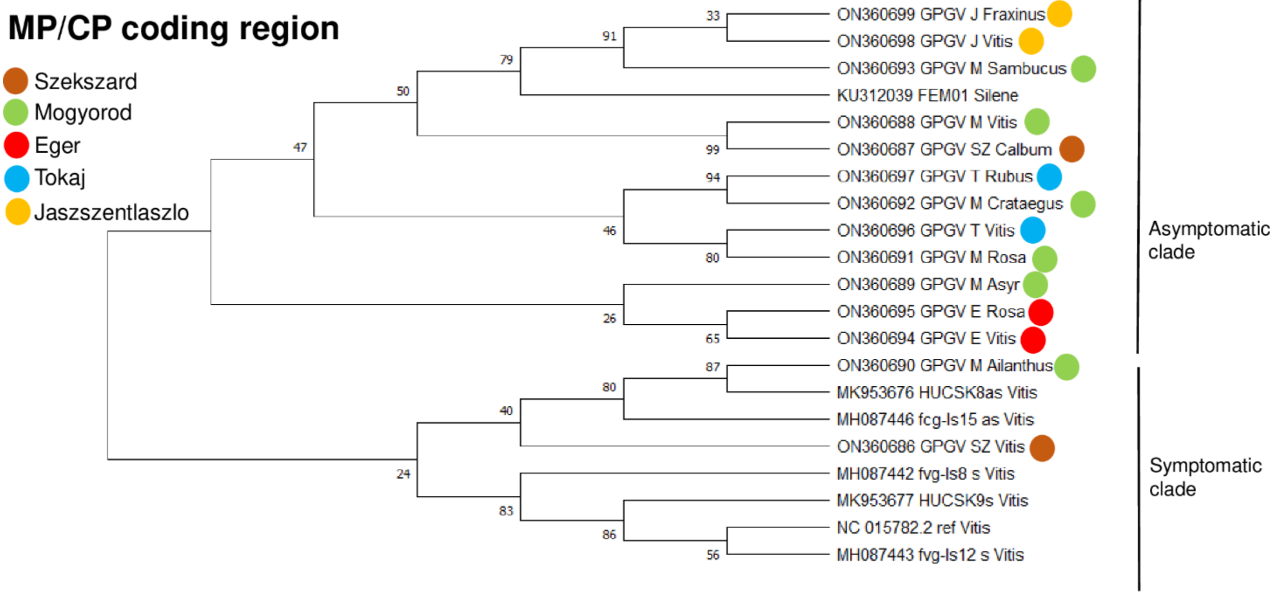

Figure S8. Evolutionary analysis of 411 bp part of MP/CP coding region of GPGV by maximum likelihood method.

The evolutionary history was inferred by using the maximum likelihood method and Tamura-Nei model [1]. The bootstrap consensus tree inferred from 1000 replicates [2] is taken to represent the evolutionary history of the taxa analysed. Branches corresponding to partitions reproduced in less than 50% bootstrap replicates are collapsed. The percentage of replicate trees in which the associated taxa clustered together in the bootstrap test 1000 replicates) are shown next to the branches [2]. Evolutionary analyses were conducted in MEGA11 [3]. Colour circles indicates the geographical origin of the strains. HUCSK8as and HUCSK9s are asymptomatic and symptomatic GPGV strains described in Hungary [4]. Ref is the GPGV reference genome from grapevine, while FEM01 strain was described from *Silene* [5]. Symptomatic (s) and asymptomatic (as) GPGV isolates Fvg-is-8_s, 12_s, 15_as were described by Tarquini et al [6].

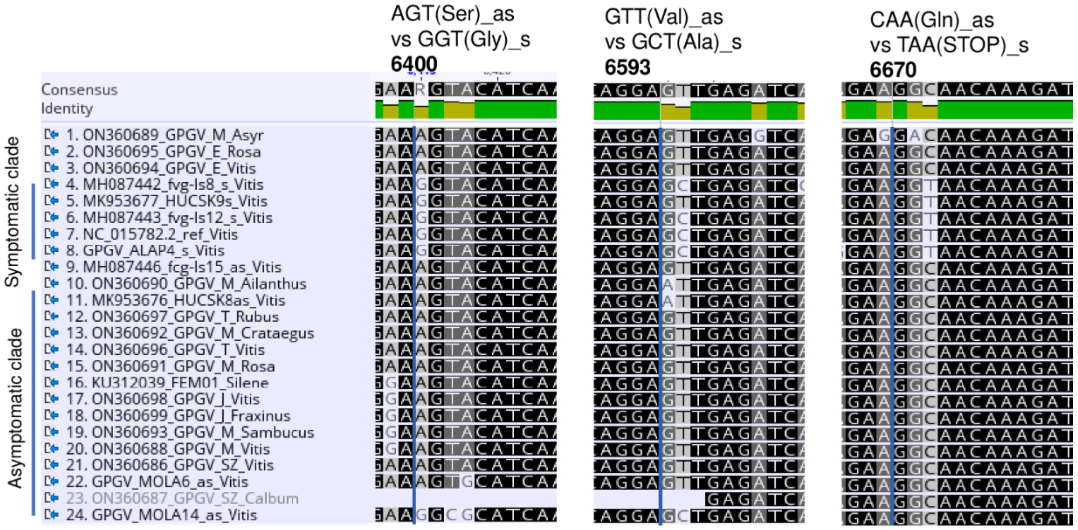

Figure S9: Polymorphism in movement coding region of asymptomatic and symptomatic GPGV variants.

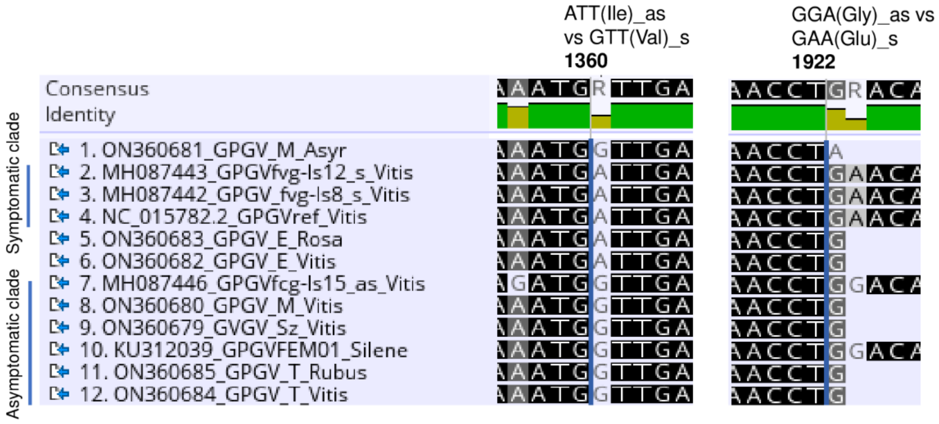

Figure S10: Polymorphism in replicase coding region of asymptomatic and symptomatic GPGV variants.

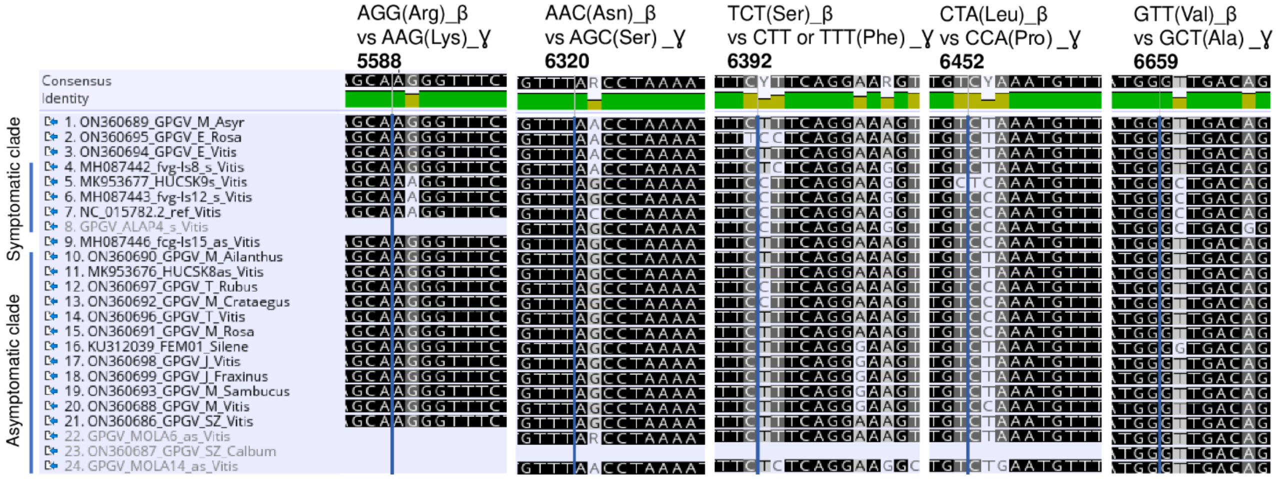

Figure S11: Polymorphism in movement protein coding region of asymptomatic and symptomatic GPGV var-iants in position differentiating between clade β and ɣ.

References:

1. Tamura, K.; Nei, M. Estimation of the number of nucleotide substitutions in the control region of mitochondrial DNA in humans and chimpanzees. *Molecular Biology and Evolution* **1993**, *10*, 512-526.10.1093/oxfordjournals.molbev.a040023
2. Felsenstein, J. Confidence limits on phylogenies: An approach using the bootstrap. *Evolution* **1985**, *39*, 783-791.10.1111/j.1558-5646.1985.tb00420.x
3. Tamura, K.; Stecher, G.; Kumar, S. Mega11: Molecular evolutionary genetics analysis version 11. *Molecular Biology and Evolution* **2021**, *38*, 3022-3027.10.1093/molbev/msab120
4. Demian, E.; Jaksa-Czotter, N.; Molnar, J.; Tusnady, G.E.; Kocsis, L.; Varallyay, E. Grapevine rootstocks can be a source of infection with non-regulated viruses. *European Journal of Plant Pathology* **2020**, *156*, 897-912.10.1007/s10658-020-01942-w
5. Gualandri, V.; Asquini, E.; Bianchedi, P.; Covelli, L.; Brilli, M.; Malossini, U.; Bragagna, P.; Saldarelli, P.; Si-Ammour, A. Identification of herbaceous hosts of the grapevine pinot gris virus (gpgv). *European Journal of Plant Pathology* **2017**, *147*, 21-25.10.1007/s10658-016-0989-4
6. Tarquini, G.; De Amicis, F.; Martini, M.; Ermacora, P.; Loi, N.; Musetti, R.; Bianchi, G.L.; Firrao, G. Analysis of new grapevine pinot gris virus (gpgv) isolates from northeast italy provides clues to track the evolution of a newly emerging clade. *Archives of Virology* **2019**, *164*, 1655-1660.10.1007/s00705-019-04241-w
