# Supplementary material for: Grapevine Pinot Gris Virus Is Present in Different Non-Vitis Hosts
Source: Plants (Basel). 2022 Jul 12;11(14):1830. doi: 10.3390/plants11141830 (PMC9315623; doi:10.3390/plants11141830)
Supplement: Supplementary file 1 [file plants-11-01830-s001.zip › Table S1.pdf]

plants MDPI

*Table S1 to*

# Grapevine Pinot gris virus is present in different non-*Vitis* hosts

**Emese Demian, Nikoletta Jaksa-Czotter and Eva Varallyay***

* Correspondence: varallyay.eva@uni-mate.hu

**Table S1**

Sequences of the used of PCR primers for virus detection with their appropriate references

| Primer Name | Primer Sequence (5'-3') | amplified product | annealing temperature | Position on the reference genome | Funcion of the amplified region | Genome used as a reference | Reference* |
|---|---|---|---|---|---|---|---|
| GPG-6609F | GAGATCAACAGTCAGGAGAG | 411 | 62 | 6610-6629 | coat protein | KU312039 | Glasa et al., 2014 |
| GPG-7020R | GACTTCTGGTGCCTTATCAC | | | 7021-7002 | | | |
| GPGV 5557F | ACTTATCTGATGGCTCTGATG | 1652 | 62 | 5569-5589 | RdRP-MP-CP-3'UTR | | Czotter et al., 2018 |
| GPGV 7220R | GTTACGTGCTCCTATGAGAC | | | 7221-7202 | | | |
| GPGV-10F | CAATTGATCCCGTGTAGTGC | 2004 | 62 | 21-40 | 5'UTR-RdRP | | this work |
| GPGV-2015R | CAGGTTCATYTTTGGATTCAAC | | | 2025-2004 | | | |
| GPGV5578_F_KpnI | caggtaccATGGCTCTGATGAAGAGGAT | 1600 | 64 | 5578-5597 | RdRP-MP-CP-3'UTR | | |
| GPGV7177_R_XbaI | tctagaCTACATACTRAATGCACTCTCC | | | 7178-7156 | | | |
| GPG-526A2 | GGATGGATGTATCTCCTGAG | only for sequencing | | 517-536 | | | |

Cycling parameters during RT-PCR reaction using NEB Q5 DNA polymerase:

| | | |
|---|---|---|
| 98◦C | 30 s | |
| 98◦C | 10 s | 40x |
| annealing temperature | 20 s | |
| 72◦C | 60 s | |
| 72◦C | 2 min | |

**References:**

**Glasa M**, Predaja L, Komnek P, Nagyov A, Candresse T, Olmos A **(2014)** Molecular characterization of divergent Grapevine Pinot Gris Virus isolates and their detection in Slovak and Czech grapevines. Arch Virol 159(8):2103–2107. https://doi.org/10.1007/s00705-014-2031-5

**Czotter N**, Molnar J, Szabó E, Demian E, Kontra L, Baksa I, Szittya G, Kocsis L, Deak T, Bisztray G, Tusnady GE, Burgyan J and Varallyay E **(2018)** NGS of Virus-Derived Small RNAs as a Diagnostic Method Used to Determine Viromes of Hungarian Vineyards. Front. Microbiol. 9:122. doi:
